# Recalcitrant vegetative and nodular scalp lesions on the vertex in pemphigus patients: an immunocompromised district?^☆^

**DOI:** 10.1016/j.abd.2022.01.007

**Published:** 2022-10-29

**Authors:** Rifkiye Kucukoglu, Tugba Atci, Goncagul Babuna-Kobaner, Nesimi Buyukbabani

**Affiliations:** a Department of Dermatology & Venereology, Istanbul Faculty of Medicine, Istanbul University, Istanbul, Turkey; b Department of Pathology, Istanbul Faculty of Medicine, Istanbul University, Istanbul, Turkey

Dear Editor,

Although scalp involvement is common in patients with pemphigus vulgaris (PV) and pemphigus foliaceus (PF), vegetative scalp lesions have rarely been reported in PV patients.^1^, ^2^, ^3^, ^4^, ^5^ Here we present two patients with mucocutaneous PV and one patient with PF who developed recalcitrant vegetative lesions and nodular lesions on the scalp during their disease course.

Among a total of 524 PV and PF patients followed up at the bullous disease outpatient clinic, only three (0.6%) developed recalcitrant vegetative and nodular lesions on the scalp (Figs Figure 1, Figure 2, Figure 5 ). The demographic, clinical, and immunopathological features and treatment characteristics of these three patients are shown in Table 1.

**Figure 1:**
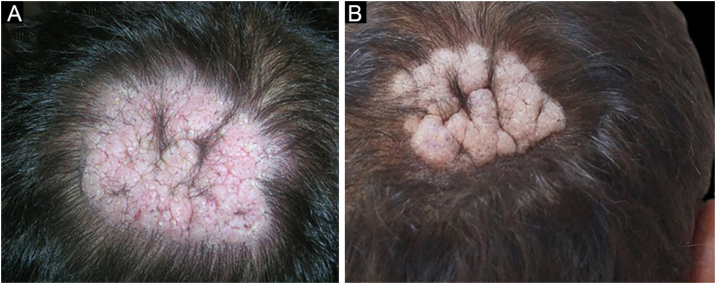
PV Patient 1. (a) An alopecic vegetative plaque with erythema, erosions, and crusting, (b) A cicatricial alopecic verrucous plaque on the right vertex after remission of the disease.

**Figure 2:**
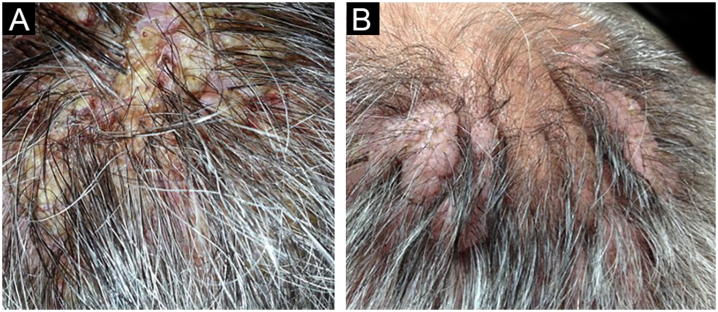
PV Patient 2. (a) Vegetative scalp plaques with oozing, crusting, erosions and fissures, (b) Lobulated verrucous plaques on the right and left vertex of the scalp after remission of the disease.

**Figure 5:**
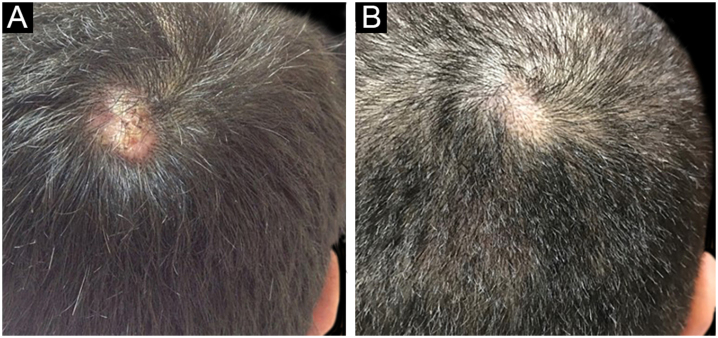
(a) An alopecic nodule on the right vertex in PF patient, (b) Almost complete regression of the nodular lesion with only minimal alopecia.

**Table 1:** Demographic, clinical, and laboratorial and treatment features of the pemphigus patients.

Variables	PV Patient 1	PV Patient 2	PF Patient 3
Age (years)/Sex	68/Male	63/Male	46/Male
Direct immunofluorescence	Intercellular deposition of IgG and C3 within epidermis	Intercellular deposition of IgG and C3 within epidermis	Intercellular deposition of IgG and C3 within epidermis
Indirect immunofluorescence	Positive	Positive	Positive
ELISA	ND	Desmoglein 3: 9.1^a^	Desmoglein3: negative
		Desmoglein 1: 5.3^a^	Desmoglein1: 4.9^a^
Diagnosis	Pemphigus vulgaris	Pemphigus vulgaris	Pemphigus foliaceus
Localization of vegetative/nodular scalp lesions	Right vertex	Right and left vertex	Right vertex
Cutaneous involvement (other than scalp)	+	+	+
Mucosal involvement	Oral	Oral and nasal	–
Treatment	SC, AZA, ILC	SC, AZA, MMF, Rituximab, IVIG, ILC	SC, AZA, ILC
Follow-up (years)	8	9	6

PV: pemphigus vulgaris; PF: pemphigus foliaceus; IgG, Immunoglobulin G; C3, Complement C3; ND; Not determined; SC, Systemic Corticosteroid; AZA, Azathioprine; ILC, Intralesional Corticosteroid; MMF, Mycofenolate Mofetil; IVIG, Intravenous Immunoglobulin; .

a Cut-off value: 1 ratio.

The first and second patients had a diagnosis of PV presenting with mucocutaneous involvement including scalp (Figs. 1,2). Four years following the diagnosis, a treatment-resistant, vegetative scalp lesion appeared on the right vertex of the first patient (Fig. 1a). The second patient also developed treatment-resistant scalp lesions, gradually becoming vegetative on the right and left vertex, two years after the diagnosis (Fig. 2a). In addition to the vegetative scalp lesions, non-compliance with therapy and frequent disease activations mostly occurring on these vegetative scalp lesions were other common features of these two patients. Despite the achievement of the clinical and immunological remission with treatment, residual vegetative masses on the scalp, associated with cicatricial alopecia in the first patient, remained in both of them (Figs. 1b, 2 b). The third patient had a diagnosis of PF presenting with cutaneous involvement including the scalp. An alopecic, nodular scalp lesion firstly appeared on the right vertex, three years following the diagnosis (Fig. 5a). At that time, he had a high level of anti-desmoglein-1 antibody titer (Table 1) without any other cutaneous involvement and the nodular scalp lesion showed a significant regression under intralesional corticosteroid treatment (Fig. 5b). Although scalp plaques were clinically vegetative in the first and second patients, histopathological examination performed at the time of disease activation in both patients revealed findings compatible with PV, rather than pemphigus vegetans (Figs Figure 3, Figure 4). Moreover, the classical intertriginous, vegetative, or papillomatous lesions of pemphigus vegetans were not observed in these two patients. Unfortunately, a histopathological examination of the scalp lesion could not be performed for PF patient.

**Figure 3:**
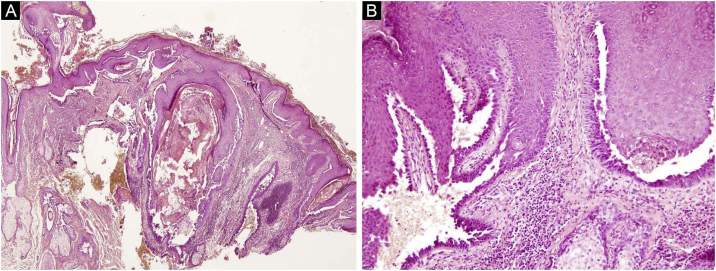
(a) The biopsy shows acanthotic epidermis, suprabasal cleavage on both epidermis and all hair follicles (Hematoxylin & eosin, ×40), (b) Epidermis and hair follicle epithelium showing suprabasal acantholysis, plasma cell-rich inflammatory infiltrate in surrounding dermis (Hematoxylin & eosin, ×200).

**Figure 4:**
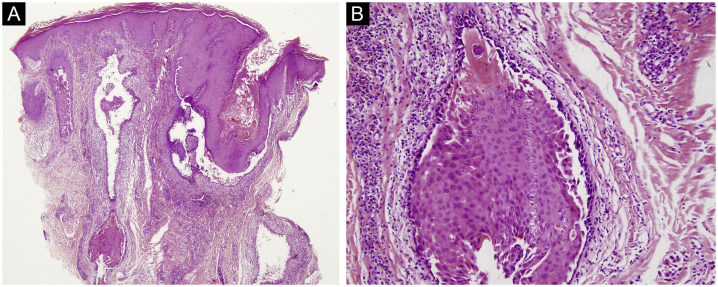
(a) Panoramic view of the biopsy reveals extensive suprabasal acantholytic cleavage in nearly all hair follicles (Hematoxylin & eosin, ×40), (b) High power view of suprabasal acantholysis in follicle epithelium, highly suggestive of pemphigus (Hematoxylin & eosin, ×200).

Scalp involvement is reported in up to 60% of pemphigus patients in various series.^1^, ^2^ However, vegetative scalp lesion has previously been reported in only three PV patients, to the best of our knowledge.^3^, ^4^, ^5^ All of the previously reported patients with vegetative scalp plaques presented as a localized form of PV, unlike the present study’s patients.^3^, ^4^, ^5^ The vegetative scalp lesions of PV patients occurred during treatment of recalcitrant disease course and particularly influenced by frequent disease activations. Additionally, to the best of our knowledge, a disease activation limited to a nodular, alopecic scalp lesion, as seen in the PF patient, has never been reported before. In both of the present study’s PV patients, residual vegetative masses remained on the scalp despite the disease remission (Figs. 1b, 2 b). On the other hand, the nodular scalp lesion in our PF patient showed almost complete regression under intralesional corticosteroid treatment (Fig. 5b).

We think that these scalp lesions which were observed during the disease course may have occurred as a result of a hypertrophic healing process of the recalcitrant pemphigus lesions. Recently, the development of keratotic verrucous plaques on the trunk was reported in a patient with long-lasting PF which was resistant to various therapies, similar to the PV patients.^6^

Interestingly, the clinical appearance of the scalp lesions in all three patients were vegetative plaques and nodular lesions mainly localized on the right vertex. The other possible mechanism for the scalp lesions in the present study’s patients may be explained by the “immunocompromised district” concept, proposed by Ruocco et al., which denotes a regional immune dysregulation characterized by either reduction or induction of immunity.^7^ One of the suggested pathophysiological mechanisms is that disruption of lymph circulation leads to trafficking of immune cells, inducing an altered immune response that can be excessive, favoring the outbreak of immune disorders.^7^ In the present study, the lymph circulation might be affected by long-lasting scalp lesions with hypertrophic healing, which might have given rise to an antigen burden on the vertex of the scalp, resulting in frequent disease activations followed by a repetitive healing process in a sort of vicious cycle. However, it is a matter of debate why these recalcitrant lesions vulnerable to disease activations were localized at similar sites in the patients.

In conclusion, the present PV and PF patients had scalp involvement with vegetative plaques and nodular lesions on the vertex, representing a distinct and rare clinical manifestation.

## Financial support

None declared.

## Authors' contributions

Rifkiye Kucukoglu: Critical literature review; data collection, analysis and interpretation; effective participation in research orientation; intellectual participation in propaedeutic and/or therapeutic management of studied cases; manuscript critical review; preparation and writing of the manuscript; study conception and planning.

Tugba Atci: Critical literature review; data collection, analysis and interpretation; effective participation in research orientation; manuscript critical review; preparation and writing of the manuscript; study conception and planning.

Goncagul Babuna-Kobaner: Critical literature review; data collection, analysis and interpretation; effective participation in research orientation; manuscript critical review; preparation and writing of the manuscript; study conception and planning.

Nesimi Buyukbabani: Data collection, analysis and interpretation; effective participation in research orientation; intellectual participation in propaedeutic and/or therapeutic management of studied cases; manuscript critical review; preparation and writing of the manuscript; study conception and planning.

## Conflicts of interest

None declared.
